# Evaluating the Safety and Effectiveness of Regional Upper GI Bleed Services Through an Outcome-Based Analysis at a Centre in the United Kingdom

**DOI:** 10.7759/cureus.74904

**Published:** 2024-12-01

**Authors:** Chaitthanya Sai, Sangeetha Baskar, Abhinav Nair, John Schembri, Srishti Bhagrav, Kushan Kumarapeli, Nilanga Nishad, Mo Thoufeeq

**Affiliations:** 1 Gastroenterology, Sheffield Teaching Hospitals NHS Foundation Trust, Sheffield, GBR; 2 Hospital Medicine, Brønderslev Psykiatriske Sygehus, Brønderslev, DNK

**Keywords:** endoscopy, haematemesis, malena, regional bleed service, upper gi bleeding

## Abstract

Background

The aim of this study was to compare the clinical characteristics and clinical outcomes of patients who presented with acute upper gastrointestinal bleeding (AUGIB) among two groups of patients who were transferred from local and district hospitals for endoscopy and subsequent management versus direct admissions to the emergency department with AUGIB to the Sheffield University Hospital NHS Trust.

Methods

We included 259 patients who underwent upper GI endoscopy from April 2018 to March 2022, of whom 29 were transferred and 230 were direct admissions. The analysis focused on demographics, pathological findings, time to endoscopy, blood transfusions, and hospital stay.

Results

The transferred patients were younger (median age 58 vs. 67 years) and received early endoscopic interventions compared to direct admissions (mean time to endoscopy 0.97 vs 2.1 days/p=0.001). The means (s.d.) of hospital stay among the transferred and direct admission groups were 9.9 (9.5) and 8.3 (9.7) days, respectively (p=0.43), regardless of intervention timing or transfusion needs. The timing of the procedure did not influence the length of hospital stay from the time of endoscopy to discharge (p=0.241). Thirty-day mortality was 8 (28%) in the transferred group and 20 (9%) in the non-transferred group (p=0.006).

Conclusion

These findings underscore that regional bleeding services are safe and effective.

## Introduction

Acute upper gastrointestinal bleeding (AUGIB) represents a significant medical emergency, with a presentation rate of one every six minutes in the United Kingdom and an annual incidence of 134 cases per 100,000 people [1]. Despite advancements in medical care, the mortality rate among new admissions with AUGIB in the UK was 7%, while among inpatients in 2007, it was 26% [2]. Recent data indicate an increasing trend in mortality rates among the US population in 2021 compared to the period from 2012 to 2019, highlighting the ongoing challenges and the need for improved management strategies for AUGIB [3].

Substantial initiatives have been undertaken to enhance both the clinical and organizational management of patients with AUGIB. The evidence concerning early endoscopy has been contentious, as certain studies have indicated that early intervention through endoscopy may not alter outcomes [4]. However, more recent research has proposed that early endoscopy could lead to reduced resource utilization, enhanced patient outcomes, and decreased mortality rates [5].

The current consensus acknowledges that the optimal standard of care for AUGIB should involve round-the-clock access to specialists in endoscopy, interventional radiology, and surgery [6,7]. Ninety percent of the hospitals provide out-of-hours (OOH) onsite endoscopy services according to the British Society of Gastroenterology (BSG) upper GI bleeding audit in 2022, which is low in comparison to the audit in 2007 (92%) [8]. According to the BSG/NHS England National Survey in 2014-2015 on the provision of OOH services for acute AUGIB in England, 80% of hospitals offer 24/7 endoscopy services for unstable patients, and an additional 10% are part of networks providing acute services; only 60% perform endoscopies within 24 hours for stable acute admissions or inpatients with AUGIB in the UK. These statistics have shown minimal changes since the last audit in the UK in 2013, with 77% providing OOH services and 56% conducting endoscopies within 24 hours [9].

Some hospitals have set up their own way of providing care by setting up methods such as inter-hospital transfer methods for acute management of AUGIB and have shown that regional bleeding services are effective and safe [10].

Sheffield University Hospital Trust provides 24/7 AUGIB services directly to its emergency department attendees with an on-call gastroenterologist. In addition, they provide 24/7 regional UGIB services to three of the region’s hospitals as a goodwill arrangement but are not commissioned and it does not have a policy on referral criteria as well. These three hospitals offer AUGIB services during daytime hours but do not provide them after 5:00 PM on weekdays or during weekends and holidays. This is the first-ever evaluation of regional UGIB services in the UK.

## Materials and methods

The objective of this study was to compare the clinical characteristics and evaluate the clinical outcomes of patients presenting with acute upper gastrointestinal bleeding (AUGIB) across two distinct patient cohorts: those transferred from regional district general hospitals for endoscopy and subsequent management, and those admitted directly to the emergency department (ED) of the Sheffield University Hospital NHS Trust with AUGIB.

Methodology

This study employed a retrospective design, analyzing data from patients referred for the management of AUGIB to the Sheffield University Hospital NHS Trust between April 2018 and March 2022. The referred patients were primarily transferred for endoscopic evaluation and further management. To facilitate a comparative analysis, data were also collected for all patients who were directly admitted to the ED of Sheffield University Hospital with AUGIB during the same period. Patient records were accessed through electronic health systems to gather information on baseline clinical characteristics (e.g., age, sex, comorbidities, and presenting symptoms), pathological diagnoses (e.g., peptic ulcer, variceal bleeding, etc.), and clinical management details. Specific parameters included the time from admission to endoscopy, length of hospital stay, and 30-day mortality rates. The collected data underwent rigorous statistical analysis to determine significant differences between the two groups. Continuous variables were analyzed using independent t-tests, while categorical data were evaluated using chi-squared tests. The analysis was performed using SPSS software (version 20, IBM Corp., Armonk, USA).

Key variables assessed

Demographics and Baseline Characteristics: Age, gender, comorbidities (e.g., chronic liver disease, cardiovascular conditions), and initial hemodynamic stability.

Pathological Diagnoses: Types and frequencies of the underlying causes of AUGIB.

Timing and Outcomes of Intervention: Time from admission to endoscopy and subsequent clinical outcomes, including length of hospital stay and mortality within 30 days post-admission.

Clinical and procedural outcomes between patients transferred from regional hospitals and those directly admitted to the emergency department were analysed.

This article was previously posted to the Research Square preprint server on 25 September 2024 [11].

## Results

During the specified time period, 29 patients were transferred and 230 patients were directly admitted for AUGIB at the two hospitals within the trust.

Table 1 presents a comparison between two groups: the "Transferred group" and the "Non-Transferred group". It compares the basic characteristics, timing of endoscopy, number of blood transfusions following admission, and length of stay between the two groups. This analysis highlights significant differences in age, timing of intervention, and blood transfusion requirements between the Transferred and Non-Transferred groups, with a tendency toward more expedited endoscopy and less blood utilization in the Transferred group. However, the length of hospital stay did not show a notable difference between the two groups. The 30-day mortality rate was greater in the transferred group (p=0.006).

**Table 1:** Comparison of basic characteristics, timing to endoscopy, blood transfusions following admission, and length of stay between the two groups

		Transferred group (n=29)	Non-Transferred group (n=230)	Significance	
						Statistic	p-value
Age (years) median (25^th^-75^th^ Percentile)		58(51.2-66.8)	66.6 (64-69.2)	t-value=1.92 (independent sample t-test)	0.037
Gender	Males	14 (48%)	154(58%)	Chi. squared value = 1.56	0.42
	Females	15 (52%)	56 (42%)		
Timing of the endoscopy					
	< 12 hours	9(31%)	56(24%)	-	-
	12-24 Hours	6(21%)	73(32%)	-	-
	24-48 hours	1(3%)	70(30%)	-	-
	>48 hours	7(24%)	31(14%)	-	-
	Not done	6(21%)	0(0)	-	-
	Mean (s.d.) hours	0.97(1.4)	2.1(3.1)	t-value=3.85 (independent sample t test)	0.001
Blood transfusion					
	0	24	121	-	-
	1 to 2	5	71	-	-
	> 3	0	38	-	-
Length of stay (days)				-	-
	25th Percentile	4	3	-	-
	50th percentile	7	5	-	-
	75th Percentile	16.5	10	-	-
	Mean(s.d.)	9.9 (9.5)	8.3 (9.7)	t- value=1.34	0.43
30-day mortality		8 (27.6%)	20 (8.7%)	t- value= 2.88	0.006

Table 2 highlights notable differences in the distribution of various pathologies between the Transferred and Non-transferred groups. The Transferred group exhibited a greater prevalence of varices and certain other pathologies than the Non-transferred group. Understanding these differences can provide insights into the characteristics and clinical profiles of patients requiring transfer versus those managed within the same facility.

**Table 2:** Diagnosis made via endoscopy

Pathology	Transferred group		Non-transferred group	
	n	%	n	%
No	5	17.2	73	31.7
Ulcer/gastric or duodenal	4	13.8	64	27.8
Severe Oesophagitis	2	6.9	18	7.8
Varices	7	24.1	16	7.0
Portal hypertensive gastropathy	0	0	11	4.8
Mallory Weiss tear	0	0	10	4.3
Angiodysplasia	0	0	7	3.0
Altered blood	0	0	6	2.6
Erosive gastritis	1	3.4	6	2.6
Duodenal tumour	0	0	4	1.7
Leiomyoma	0	0	3	1.3
Dieulafoy’s	0	0	2	0.9
Oesophageal polyp/lesion	0	0	2	0.9
Gastric bypass bleed	0	0	2	0.9
Gastric antral cascular ectasia	0	0	1	0.4
Gastric varices	1	3.4	1	0.4
Duodenal fistula	0	0	1	0.4
Gastrointestinal stromal tumours	0	0	1	0.4
Post oesophageal endoscopic mucosal resection (EMR)	0	0	1	0.4
Sphincterotomy bleed	0	0	1	0.4
Banding ulcers	2	6.9	0	0.0
Percutaneous endoscopic gastrostomy site bleeding	1	3.4	0	0.0
No scooping done	6	20.7	0	0.0
Total	29	100	230	100.0

A significant percentage (n=6/20.7%) of patients in the Transferred group and the Non-transferred group were not scoped. Among them, two patients were directly examined with small bowel video capsular endoscopy, one patient who worsened during endoscopy and died (those with decompensated cirrhosis and frailty), one patient who was offered transjugular intrahepatic portosystemic shunt (TIPS), and two patients who did not need scoping. The details of the patients who died after being transferred for AUGIB are described in Table 3.

**Table 3:** Patient details who died after transfer for management of acute UGIB *Cause of death - Multi-organ failure and spontaneous upper gastrointestinal bleeding ARLD - alcohol-related liver disease, AF - Atrial fibrillation, TIPS - Transjugular intrahepatic portosystemic shunt, UGIE - Upper GI endoscopy, CABG - Coronary artery bypass grafting, COPD - Chronic obstructive pulmonary disease, ERCP - endoscopic retrograde cholangiopancreatography, DNA-CPR - do not attempt cardiopulmonary resuscitation

Age	Gender	Co-morbidities	Duration to endoscopy	Intervention	Time to death since admission
(years)					
65	Male	Cirrhosis, Renal failure, Variceal bleeding with banding ulcers	3 Hours	Danis stent and TIPS (day 5)	Day 12
80	Female	Congestive cardiac failure and atrial fibrillation on long-term anticoagulation, after ERCP	9 hours	Found to have a large clot over the papilla, clot removed.	Day 1
43	Male	ARLD with decompensated cirrhosis and chronic pancreatitis.	Not done	Patient was not stable for UGIE. Haemoglobin (Hb) was 44.	Day 4
89	Female	AF and heart failure and diabetes mellitus, and on long-term anticoagulation	Not done	Small bowel capsular endoscopy planned/but not done	Day 4
62	Female	ARLD, acute renal failure, septicaemia, and shock (DNA-CPR)	8 days	Varices banding	Day 10
59	Male	ARLD and decompensated cirrhosis	12 hours	Rescoped in 2 days due to continuous malena and dropping Hb. Scoped with paediatric colonoscope duodenitis. No intervention done.	Day 6*
79	Female	Chronic kidney disease, ischemic heart disease with CAGB 3 times had an ERCP for cholangitis	Not done	Angiographic intervention was done for duodenal bleeding on day 3	Day 20
62	Male	Large mediastinal non-Hodgkins lymphoma and COPD, pulmonary embolism, and acute copulmonale. Suspected small intestinal bleed	Not done	Small bowel capsular endoscopy and vascular angiography done on 11th day	Day 18

## Discussion

This study highlights that regional upper gastrointestinal bleeding (UGIB) services can be safe and effective. It is the first study to specifically illustrate the operational structure and outcomes of a regional UGIB service within the UK. This demonstrates that a safe and effective service can be delivered through a well-coordinated clinical team at a larger receiving hospital. The success of such a service relies on the commitment and enthusiasm of the clinical team at the tertiary center. However, the service is currently not commissioned or job-planned for consultants at the tertiary center, which raises concerns about long-term sustainability. Planning such services within job roles could enhance sustainability and minimize the risk of burnout among healthcare providers - a concern increasingly recognized in emergency care and other high-demand medical specialties [12].

Transfer protocols and patient care outcomes

The study's findings emphasize the importance of an agreed-upon transfer policy, ensuring timely transfers from district general hospitals (DGHs) without local gastroenterology services. Upon arrival at the Northern General Hospital, patients were reassessed by the emergency care team in conjunction with the on-call gastroenterologist. Such coordinated management aligns with national and international guidelines recommending early endoscopic intervention for acute UGIB to improve outcomes and reduce mortality rates [13]. The use of severity scores such as Rockall will be useful in determining the appropriateness of referrals and resource allocation.

The comparison between transferred and non-transferred patients undergoing endoscopy for acute UGIB revealed significant differences in demographic and clinical characteristics, which carry implications for patient care. For example, patients in the transferred group were significantly younger, with a lower median age. This aligns with prior research suggesting that younger patients may experience more acute or severe bleeding episodes necessitating specialized care [14-15]. However, it is well-documented that elderly patients, due to comorbidities and frequent use of anticoagulants, are at higher risk of mortality and complications from UGIB. [16-17]. While there was no statistically significant difference in sex distribution, the role of underlying comorbidities in influencing clinical outcomes should not be overlooked [18-19].

Clinical interventions and blood transfusion

The promptness of intervention in the transferred group underscores the importance of regional systems that enable timely endoscopic intervention. Early endoscopy within 24 hours is associated with better outcomes, as supported by various guidelines [2,20]. Interestingly, the Transferred group required fewer blood transfusions, possibly because transfusions were already administered as part of the initial resuscitation at the referring DGH. While detailed data on pre-transfer resuscitation were not available, this finding highlights the potential for effective local stabilization to improve outcomes.

Lower transfusion requirements could also reflect the success of targeted endoscopic interventions to control bleeding sources more effectively, which reduces the need for subsequent transfusions. This aligns with studies emphasizing that early and targeted interventions can decrease the overall transfusion burden in patients with UGIB [21].

Hospital stay and mortality

The study found no significant difference in the length of hospital stay between groups. However, the higher mean number of days and the 75th percentile in the transferred group suggest a clinically important impact on bed occupancy at the tertiary center. This finding emphasizes the need to balance effective care delivery with resource allocation in receiving hospitals.

The 30-day mortality rate was higher in the transferred group, which could be attributed to greater comorbidities. This aligns with the understanding that patients with multiple comorbid conditions face poorer outcomes following UGIB, particularly in the context of delayed intervention or complex clinical profiles [16].

Training and skills retention in DGHs

An important concern raised by this study is the potential for de-skilling among clinical and nursing teams at DGHs due to the lack of out-of-hours (OOH) UGIB services. While only a small percentage of patients require endoscopy immediately after resuscitation, the absence of OOH endoscopy services limits the ability to provide urgent endoscopy within the recommended 2-hour window following optimal resuscitation, as highlighted by NCEPOD (National Confidential Enquiry into Patient Outcome and Death) recommendations [22]. Maintaining OOH services at DGHs may help preserve critical skills among healthcare teams and enhance local care delivery.

Study limitations

The study has several limitations that warrant acknowledgment. First, there was a lack of detailed information on the care provided at base hospitals before transfer, including medications administered, resuscitation strategies, and transfusion volumes. This limits the ability to fully evaluate the impact of pre-transfer care on clinical outcomes. Additionally, the study did not capture data on patients deemed unfit for transfer, which could introduce selection bias and underestimate the true burden of UGIB requiring tertiary care.

## Conclusions

Emergency services should have access to 24/7 endoscopy to look after patients presenting with upper GI bleed. However, challenges exist in providing these services in peripheral hospitals that do not have round-the-clock services. We found that the regional upper GI bleed service may be safely and effectively carried out and services should ensure that a sustainable practice prevails by having policies and job planning arrangements in place. 

This has illustrated that a safe and effective service can be delivered if there is a willing and keen clinical team in the larger teaching hospitals. Since the services are currently run by goodwill arrangements, policy-based arrangements are recommended.
